# The Student's Medical Dictionary

**Published:** 1897-09

**Authors:** 


### Reviews of Books.

**The Student's Medical Dictionary.** By GEORGE M. GOULD, M.D. Pp. xii., 17—701. Tenth Edition. London: H. K. Lewis. 1896.

After making allowance for some of the eccentricities of spelling and pronunciation in which the people of the States indulge, we can confidently recommend this well-known and most deservedly useful book. Though, as the author tells us, it is practically a new book, the contents have not suffered, and it will be found to be almost as useful as the larger dictionary by the same author. We are inclined to think that the definitions of the prefixes and suffixes used in chemistry might be a little fuller. For instance, -ate signifies rather more than "any salt formed by an acid acting on a base," and the suffix -ite is not mentioned. Gypsum is not $CuSO_4$. We should have been glad if Dr. Gould had given us a table of Eponymic Structures as well as the very interesting table of "Eponymic Signs and Symptoms," in which, however, we should prefer to see the possessive case of the proper name used only in those instances in which the sign or symptom is indicated, and the nominative employed when a particular part of the body is mentioned. Thus we do not think Dr. Gould is correct in speaking of "Hutchinson's teeth," but we should be with him in writing "Argyll Robertson pupil," and "Romberg's symptom." In Dr. Gould's table "Erb's palsy" finds no place, although it appears in the body of the work. "Nephropexy" is not to be found, although Dr. Gould gives "hysteropexy." We are glad to notice that in this work 1 minim is given as the equivalent of ·061 cubic centimetre instead of the incorrect ·016 in the author's larger dictionary. The blemishes to which we have called attention are, however, comparatively unimportant, and taken altogether the work is really a very complete and valuable one.

